# Suicidality among inpatients - Right under our noses

**DOI:** 10.1192/j.eurpsy.2023.2352

**Published:** 2023-07-19

**Authors:** A. S. Morais, F. Martins, V. Henriques, P. Casimiro, N. Descalço, R. Diniz Gomes, N. Cunha e Costa, S. Cruz

**Affiliations:** 1 Psychiatry, Hospital Garcia de Orta, Lisbon, Portugal

## Abstract

**Introduction:**

An inpatient suicide is a tragic event that, despite not very prevalent, should not be overlooked. It occurs in 250 in 100 000 psychiatric hospital admissions (which represents a suicide risk fifteen times greater than general population) and in 1.7-1.9 in 100 000 in general hospitals (4-5 times greater risk). Together they constitute 5-6% of all suicides.

**Objectives:**

The purpose of the authors is to explore the epidemiology, the risk factors and the prevention of suicide in inpatient setting.

**Methods:**

A brief non-systematized review is presented, using the literature available on PubMed and Google Scholar.

**Results:**

The risk was higher at admission (first week) and immediately after discharge (first 24 hours, up to two weeks).

It was found to be correlated to pour staffing, an increased number of patients with severe mental illnesses and accessibility to lethal means. Many risk factors were identified, some of them specific to context. Risk Factors at admission in a psychiatric hospital – personal or familiar suicide history, schizophrenia or mood disorder, alcohol use, involuntary admission, living alone, absence from the service without permission. Later till discharge - personal suicide history (or attempts after admission), relational conflicts, unemployment, living alone, lack of discharge planning and lack of contact in the immediate post-discharge period. In General Hospitals – chronicity and severity of somatic disease, poor coping strategies, psychiatric comorbidities and lack of liaison psychiatry.

Strategies to prevent inpatient suicide should take in environmental modification (specific to environment and specific to patient – as planned levels of supervision), optimisation of the care of the patients at suicidal risk, staff education and involvement of families in care. There are few studies on the efficacy of pharmacotherapy on reducing suicidal ideation in inpatients (just for clozapine and ketamine); some psychotherapies show promising results. The post-suicide approach cannot be neglected, whether in supporting the family, the team involved and even other patients.

**Conclusions:**

The assumption of the predictive and preventive value of the risk assessment has been under scrutiny. Depressed mood and a prior history of self-harm are well-established independent risk factors for inpatient suicide; however they lose their predictive value due to their high prevalence. Up to 70% of inpatients who committed suicide didn’t express suicidal ideation on the previous interviews. Most effective measures to prevent suicide are environmental modifications and staff education approaches, giving appropriate responses to each patient’s circumstances.

There is a paucity of literature on suicide in this setting. It should become a priority in national programs of Suicide Prevention.

**Disclosure of Interest:**

None Declared

